# Protocol for the isolation, sequencing, and analysis of the gut phageome from human fecal samples

**DOI:** 10.1016/j.xpro.2022.101170

**Published:** 2022-02-09

**Authors:** Shirley Bikel, Luigui Gallardo-Becerra, Fernanda Cornejo-Granados, Adrian Ochoa-Leyva

**Affiliations:** 1 Departamento de Microbiologia Molecular, Instituto de Biotecnologia, Universidad Nacional Autonoma de Mexico, Avenida Universidad 2001, Cuernavaca, Morelos 62210, Mexico

**Keywords:** Bioinformatics, Sequence analysis, Health Sciences, Sequencing, Metabolism, Microbiology, Microscopy, Molecular Biology, Systems biology

## Abstract

The phage-bacteria interactions in the gut microbiome are critical for health and disease, but viruses of the human gut microbiome are poorly understood. Here, we present a simple and cost-efficient protocol for collecting viral-like particles (VLPs) from human fecal samples. We describe VLPs quantification using epifluorescence and TEM microscopy, followed by DNA sequencing and bioinformatics analysis. This protocol characterizes the gut phageome in normal-weight and obese children with metabolic syndrome. It is also suitable to conduct high-throughput studies for other diseases.

For complete details on the use and execution of this profile, please refer to Bikel et al. (2021).

## Highlights

• Protocol for collecting, sequencing, and bioinformatics of viral-like particles (VLPs)
• Simple, reproducible, and cost-efficient protocol to characterize the VLPs of phages
• Revealing the number of VLPs in human fecal samples by epifluorescence and TEM microscopy
• Revealing bacteriophage abundance with functional and taxonomical analysis of VLPs

## Graphical abstract

**Figure:**
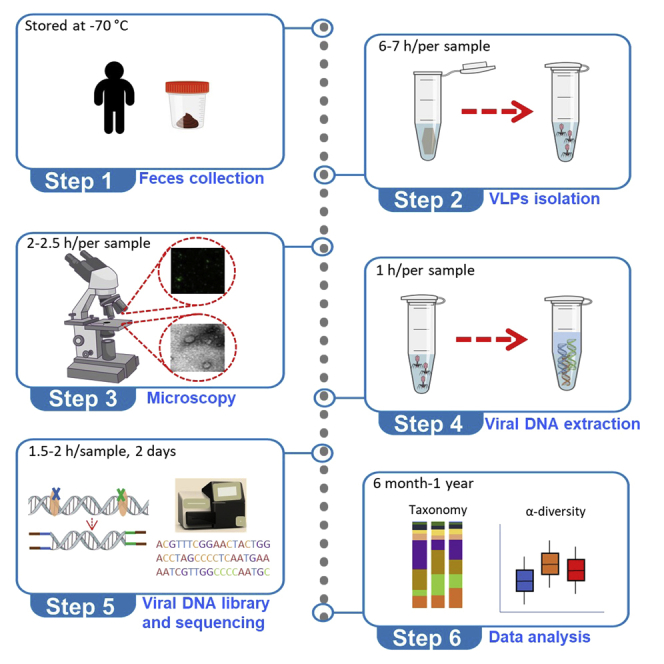

## Before you begin

The protocol below has been optimized for studying the dsDNA gut virome. It describes the specific steps to isolate virus-like particles (VLPs) from human fecal samples, sequence their DNA, and perform the bioinformatics analysis. The optimized protocol is based on the Qiamp MinElute Virus Spin kit (Qiagen) for viral DNA isolation and the Nextera XT DNA Library Preparation Kit (Illumina) for sequencing, which supports ultra-low DNA input, avoiding biases in the following quantification analysis. We recommend reading the original protocols before following the optimized protocol below.

### Fecal sample collection and storage

**Timing: 24–48 hper sample**

1. Collect fecal samples at home in a sterile cup and keep refrigerated at 4°C.
2. Transport the samples to the research facility within 12 h in a portable cooler with ice packs to preserve the temperature.
3. At the research center, aliquot samples into 250 mg portions in sterile plastic containers, resuspend in a 5 × volume with RNAlater, and store at −70°C until processing.

***Note:*** Collect samples in RNAlater to perform complementary metatranscriptome studies described in Gallardo-Becerra et al. (2020); it is not essential for virome studies.

***Note:*** The sample used in this protocol was previously reported in the Bikel et al. (2021) study. The Ethics Committee of the Instituto Nacional de Medicina Genómica (INMEGEN) in Mexico City, Mexico, approved its use. Each child's parents or legal guardians signed the informed consent form for participation, and all children assented to participate.

## Key resources table

**Table:** 

REAGENT or RESOURCE	SOURCE	IDENTIFIER
**Biological samples**		

Human fecal sample	Normal weight 9 year old female	NW_118

**Chemicals, peptides, and recombinant proteins**		

RNAlater	Thermo Fisher Scientific	Cat. AM7020
SM Buffer	G-Biosciences	Cat. N° 786-492
DNaseI	Thermo Fisher Scientific	Cat. 18047019
32% (wt/vol) paraformaldehyde	Electron Microscopy Sciences	Cat. 15714
SYBR Green	Thermo Fisher Scientific	Cat. K0221
Ampure XP Beads	Beckman Coulter	Cat. A63881

**Critical commercial assays**		

QIAamp MinElute Virus Spin Kit	QIAGEN	Cat. 57704
Qubit	Thermo Fisher Scientific	Cat. Q32851
Illumina Nextera XT DNA Library	Illumina	Cat. FC-131-1024
Bioanalyzer High Sensitivity DNA Analysis	Agilent Technologies	Cat. 5067-4626

**Deposited data**		

Assembled contigs	(Bikel et al., 2021)	BioProject PRJNA646512 BioSample SAMN15545081
In-house scripts describing the data analysis process	(Bikel et al., 2021)	https://github.com/lab8a/2021-iScience-Phageome ; in Zenodo at https://doi.org/10.5281/zenodo.5846703.

**Software and algorithms**		

FastQC	The Babraham Institute	https://www.bioinformatics.babraham.ac.uk/projects/fastqc/
Trim Galore 1.12	The Babraham Institute	https://github.com/FelixKrueger/TrimGalore
Fastx Toolkit 0.7	Hannon Lab	http://hannonlab.cshl.edu/fastx_toolkit/index.html
CD-HIT 4.6	(Fu et al., 2012)	http://cd-hit.org/
R 3.6.2	N/A	https://www.r-project.org/
IDBA-UD assembler 1.1.1	(Peng et al., 2012)	https://i.cs.hku.hk/∼alse/hkubrg/projects/idba_ud/
Bowtie 2.3.5	(Langmead and Salzberg, 2012)	http://bowtie-bio.sourceforge.net/bowtie2/index.shtml
VirSorter 2.2.1	(Guo et al., 2021)	https://github.com/jiarong/VirSorter2
QIIME 1.9	(Caporaso et al., 2010)	http://qiime.org/
DESeq2 3.13	(Love et al., 2014)	https://bioconductor.org/packages/release/bioc/html/DESeq2.html
Frag Gene Scan 1.31	(Rho et al., 2010)	https://sourceforge.net/projects/fraggenescan/
Meta Genome Analyzer 6.18.3	(Huson et al., 2018)	https://software-ab.informatik.uni-tuebingen.de/download/megan6/welcome.html
BWA aligner	(Li and Durbin, 2010)	https://github.com/lh3/bwa
seqtk	GitHub	https://github.com/lh3/seqtk
blast	(Altschul et al., 1990)	https://blast.ncbi.nlm.nih.gov/Blast.cgi?CMD=Web&PAGE_TYPE=BlastDocs&DOC_TYPE=Download
MEGAN6	(Huson et al., 2007)	https://software-ab.informatik.uni-tuebingen.de/download/megan6/welcome.html
MEGAN6 database for LCA analysis (released 2019)	National Center for Biotechnology Information	https://software-ab.informatik.uni-tuebingen.de/download/megan6/welcome.html
Homo Sapiens reference genome GRCh38.p13	Genome Reference Consortium	GenBank GCA_000001405.28
Non-redundant bacteria RefSeq database	National Center for Biotechnology Information	https://ftp.ncbi.nlm.nih.gov/refseq/release/bacteria/
Non-redundant viral RefSeq database	National Center for Biotechnology Information	https://ftp.ncbi.nlm.nih.gov/refseq/release/viral/
FIJI	(Schindelin et al., 2012)	https://imagej.net/software/fiji/
Prokaryotic Virus Orthologous groups (pVOGs)	(Kristensen et al., 2013)	https://ftp.ncbi.nlm.nih.gov/pub/kristensen/pVOGs/home.html

**Other**		

Local–Memory: 8GB required, 16GB recommended; Processors: 1 required, 6 recommended.	N/A	N/A
Computational Cluster – Memory: >64GB recommended; Processors: >8 recommended for parallel processing and large datasets	N/A	N/A
DynaMag™-2 Magnet	Thermo Fisher Scientific	Cat. 12321D
0.45 μm PES filter	Nalgene	Cat. 725-2545
0.22 μm PES filter	Nalgene	Cat. 725-2520
Amicon Ultra-15 filter unit 100 kDa	Millipore	Cat. UFC910024
SimpliAmp™ Thermal Cycler	Applied Biosystems	Cat. A24811
Multi-photonic confocal microscope	Olympus	Model FV1000
Transmission Electron Microscope	JEOL	ARM-200F

## Materials and equipment

The essential equipment of a molecular microbiology laboratory is also needed, including a conventional benchtop microcentrifuge, a vortex mixer, an electrophoresis chamber, and a laminar airflow cabinet. Also, expendable materials, including pipette tips, microcentrifuge, and PCR tubes, are necessary. In this protocol, the Applied Biosystems SimpliAmp™ Thermal Cycler was used for the library index PCR.

## Step-by-step method details

### Viral-like particles (VLPs) isolation

**Timing: 6–7 hper sample**

This step is helpful to isolate and concentrate the VLPs from the fecal samples.

Before beginning the experiments, thaw the fecal samples on ice. Also, it is essential to filter all reagents through sterile syringe filter polyethersulfone membrane (PES) units. We recommend using a 0.45 μm pore size to remove larger cell debris followed by a 0.22 μm pore size to remove remaining cell debris and bacterial-size particles. This two-step filtration protocol applies to all reagents and the supernatant of the fecal samples.

1. Preparation of phage suspension (15 min per sample).
  a. Pour 40 mL of SM Buffer, previously filtered, into a 50 mL centrifuge tube.
  b. Gently pipet the 250 mg of fecal sample and resuspend in RNA later several times until obtaining a homogeneous mixture.
  c. Add the whole RNA later + fecal sample mixture to the SM Buffer tube.

2. Remove bacterial debris (40 min).
  a. Centrifuge at 4700 g for 30 min at 4°C.
  b. Carefully transfer the supernatant (it contains the VLPs) to a 5 mL syringe attached to a 0.45 μm PES filter, using a micropipette. Filter the supernatant and collect the filtrate into a new 50 mL centrifuge tube.
    ***Note:*** When taking the supernatant with the micropipette, make sure not to disturb the pellet. We recommend using a fine tip and removing the volume very slowly.
  c. Filter all the supernatant obtained from the previous step through a 0.22 μm PES filter.
    ***Note:*** Regularly change the filters to avoid clogging them with cell debris that was not removed by the centrifugation step.
    ***Note:*** Do not push too hard with the syringe to avoid breaking the filter.
    **Pause Point:** The filtered buffer with VLPs can be stored at 4°C until needed.
    ***Note:*** Some reports have shown good stability of phages when stored at 4°C in SM buffer for more than six months (Jepson and March, 2004; Jończyk et al., 2011). Similarly, storing at 2°C–5°C has shown no significant reduction in phage titer (5%–10%) after six months (Mullan 2001).
    ***Note:*** Check that the SM buffer contains gelatin. It stabilizes VLPs membranes during storage.

3. Enrichment and washing the VLPs (2–3 h).
  a. Add 15 mL of the previous buffer with VLPs into an Amicon Ultra-15 centrifugal filter (100 KDa).
  b. Centrifuge at 5000 g in a fixed-angle centrifuge for 15–60 min at 4°C, until the remaining volume reaches ∼200 μL on the top of the Amicon filter unit.
  c. Discard the flow-through and add fresh filtrate again to the top of the filter unit.
  d. Repeat steps a, b and c until the entire buffer is filtered.
  e. Add 5 mL of SM Buffer to the top chamber of the Amicon and spin at 5000 g at 4°C for 2–5 min to wash the VLPs until the remaining volume reaches ∼200 uL on the top of the Amicon filter unit. Repeat this VLPs washing step (at least five times) until the buffer containing the VLPs is clear.
    ***Note:*** Wash several times the VLPs to diminish cellular debris that could inhibit the nucleic acid extraction and their visualization in the microscope. Eight washes of the VLPs allow a more precise visualization of viral particles by electron microscopy (TEM).
  f. Discard the collector tube of the Amicon filter unit and pipet the ∼200 μL of VLPs several times to resuspend the VLPs retained on the filter walls.
  g. Transfer the total volume ∼200 μL containing the VLPs to a 1.5 mL microfuge tube.
    ***Note:*** If the volume in the Amicon filter unit is less than 200 μL, adjust the volume using SM buffer.
    **Pause Point:** This 200 μL of concentrated VLPs in SM buffer can be stored at 4°C until needed.
    ***Note:*** Check that the SM buffer contains gelatin. The gelatin in SM buffer stabilizes VLPs membranes during storage.

4. Eliminate the remaining bacterial and human cell debris (15 min).
  a. Add 40 μL of chloroform to the 200 μL of VLPs and incubate at room temperature (20°C–25°C) for 10 min.
  b. Centrifuge at room temperature for 5 min at 20,000g to separate the aqueous phase. Take the aqueous phase carefully (it contains the VLPs) with a micropipette and transfer it to a new 1.5 mL microfuge tube.

5. Remove residual bacterial and human DNA (3 h).
  a. Eliminate the Non-virus-protected DNA by adding 2.5 units of DNase I per milliliter of VLPs, and incubate at 37.8°C for two hours, following the manufacturer’s procedure.
  b. Inactivate the DNase at 65°C for 10 min.
  c. Cool down the reaction on the laboratory table until room temperature (20 min).
    **CRITICAL:** Do not inactivate the DNase at 75°C; this temperature can damage the VLPs.
    ***Note:*** Wash two times the VLPs to clean up remnants of the DNAse reaction.
  d. Transfer the previous reaction into an Amicon Ultra-15 centrifugal filter (100 KDa) and add 15 mL of SM buffer.
  e. Centrifuge at 5000 g in a fixed-angle centrifuge for 15–60 min at 4°C, until the remaining volume reaches ∼200 uL on the top of the Amicon filter unit.
  f. Discard the flow-through, add fresh SM buffer to the top of the filter unit, and repeat step e.
  g. Discard the collector tube of the Amicon filter unit and pipet the ∼200 μL of VLPs several times to resuspend the VLPs retained on the filter walls.
  h. Transfer the total volume ∼200 μL containing the VLPs to a 1.5 mL microfuge tube.
    **Pause Point:** The VLPs can be stored at 4°C until needed.
    ***Note:*** At this point it is recommended to use TEM microscopy to confirm the presence of VLPs and count the number of particles using fluorescence microscopy.

### Microscopy visualization and VLPs counts

**Timing: 2–2.5 hper sample**

This step is to corroborate the presence and quantify the VLPs in the samples.

**Figure 1:**
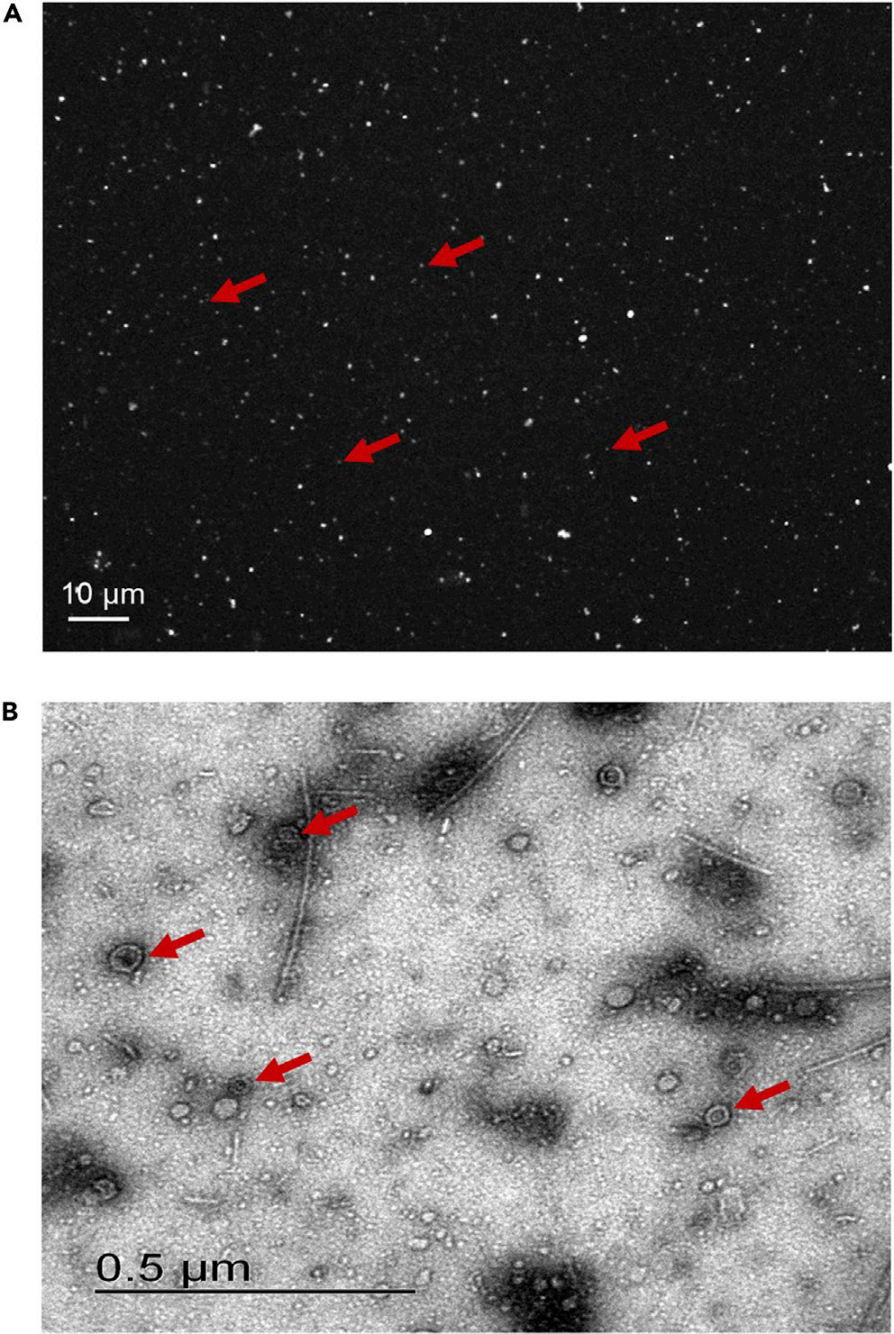
Microscopy visualization and counting of VLPs The image was modified from Bikel et al. (2021). (A) SYBR Green I-stained virus-like particles (VLPs) by epifluorescence microscopy. Red arrows show an example of the VLPs. The scale bar represents an approximate size of 10 µm. (B) TEM microscopy of VLPs. Red arrows show an example of the VLP morphology. The scale bar represents an approximate size of 0.5 µm.
    iii. Use the FIJI software (Schindelin et al., 2012) for image analysis. Any image analysis program can be used.
    iv. Count the number of fluorescents VLPs from 5 random fields per slide by triplicate and average. Calculate the absolute VLPs numbers based on the average VLPs counts per field, taking into account the area of one field, and back calculating based on the dilution factor, as follows:

6. Quantify the number of isolated VLPs by epifluorescence microscopy (1.5 h).
  a. Preparation of the SYBR Green staining solution (5 min).
    i. Dilute 5 μL of SYBR Green in 45 μL of nuclease-free water (previously passed through the 0.22 μm PES filtration units).
  b. Staining the VLPs of the sample (10 min).
    i. Place 10 μL of the VLPs sample in a 0.6 mL Eppendorf tube.
    ii. Add 2 μL of the SYBR Green solution.
    iii. Add 10 μL of 4% (wt/vol) paraformaldehyde.
      **CRITICAL:** Paraformaldehyde is a toxic chemical, and it only should be used in a chemical fume hood. Waste should be collected and discarded appropriately.
  c. Mounting the VLPs sample on a microscopy slide (5 min).
    i. Place 10 μL of stained VLPs sample on a slide.
    ii. Place a coverslip over the sample, avoiding the formation of air bubbles.
  d. Microscopy visualization and quantification of VLPs (Olympus FV1000 Multi-photonic confocal microscope) (1h).
    i. Place the slide under the microscope and focus on the field at the confocal microscope. Set the laser’s parameters to the optimal setup.
    ii. Take five micrographs of the sample, each in triplicate. This protocol uses 0.212 × 0.212-millimeter images (Figure 1A).

**Table:** 

Average number of VLP's for sample	Number of VLP's for 10 uL of sample	VLP's per 250 mg of feces (200 uL of VLP's)	VLP’s per gram of feces
P	X	Y	Z

$$X\;=\;\frac{\;p}{\;a}\;\left(b\right)$$

p = number of VLPs per sample

a = square area for the observed field in the microscope

b = total loaded area with VLPs on the microscope slide

$$Y\;=\;\frac{\;X}{\;c}\;\left(d\right)$$

c = volume loaded into slide

d = total volume of extracted VLPs for 250 mg of feces

Z = (Y)(4)

***Note:*** The optimal microscope setup depends on the microscope type and operating system.

***Note:*** Fluorescence is not stable over time. Thus the images should be taken immediately after slide preparation.

***Note:*** Some dyes can stain the phage capsid rather than staining the DNA inside it. Both stains can be used depending on the experimental need. Keep in mind that the SYBR stain does not modify the exterior of the phage.

***Note:*** It is expected to obtain between 10^8^ and 10^10^ VLPs /g in stools (Kim et al., 2011; Hoyles et al., 2014; Shkoporov et al., 2019; Bikel et al., 2021). In our hands, we obtained from 8.5 × 10^8^ to 4.8 × 10^10^ VLPs per gram of feces (Bikel et al., 2021).

7. Corroborate the phage morphology using Transmission Electron Microscopy (TEM) (1 h).
  a. Place 8 μL of a concentrated VLP sample onto carbon-coated Formvar grids.
  b. Incubate at room temperature for 1 min.
  c. Drain off the sample excess of the grid with the aid of filter paper.
  d. Stain the grids with 8 μL of 2% uranyl acetate.
  e. Incubate at room temperature for 2–3 min.
  f. Drain off the excess stain with the aid of filter paper.
  g. Dry the grids at room temperature until analysis.
  h. Take the image from the grids at magnifications of 14,000× to 80,000× (Figure 1B).

**CRITICAL:** The image fields could be obstructed in a few instances by extensive, amorphous, dark-staining material. (See potential problem 1 in the troubleshooting section).

### Viral DNA extraction and purification

**Timing: 1 hper sample**

In this step, the DNA from the isolated VLPs is extracted following the QIAamp MinElute Virus Spin Kit Handbook (https://www.qiagen.com/us/resources/resourcedetail?id=8798cda6-4c55-4c0e-a302-966521c81aec&lang=en). The QIAamp MinElute Virus Spin procedure comprises four steps (lyse, bind, wash, and elute). Perform this step using QIAamp MinElute columns in a standard microcentrifuge. This procedure is optimized for use with a starting volume of 200 μL of VLPs containing liquid.

8. Preparation of QIAGEN Protease.
  a. Add 1.4 mL of AVE Buffer to the vial of lyophilized QIAGEN Protease, and mix carefully to avoid foaming.
  b. Make sure to dissolve the QIAGEN Protease completely.
  c. Label and store the resuspended QIAGEN Protease at 2°C–8°C, only for 12 months or until the kit’s expiration date.

***Note:*** This kit provides two alternative buffers for dissolving QIAGEN Protease — Buffer AVE (recommended) or Protease Resuspension Buffer. We recommend using the AVE Buffer.

***Note:*** Storage at –20°C will prolong its life, but avoid repeated freezing and thawing. Dividing the solution into aliquots and freezing at –20°C is recommended.

9. Preparation of Buffer AW1.
  a. Add 25 mL of ethanol (96%–100%) to a bottle containing 19 mL of Buffer AW1 concentrate, as described on the bottle.
  b. Tick the check box on the label to indicate that ethanol has been added.
  c. Store reconstituted Buffer AW1 at room temperature (15°C–25°C).

***Note:*** Reconstituted Buffer AW1 is stable for up to 1 year or until the kit expiration date when stored at room temperature.

***Note:*** Always mix reconstituted Buffer AW1 by shaking before starting the procedure.

**CRITICAL:** Buffer AW1 contains chaotropic salt. Take appropriate laboratory safety measures and wear gloves when handling. Not compatible with disinfectants containing bleach.

10. Preparation of Buffer AW2
  a. Add 30 mL of ethanol (96%–100%) to a bottle containing 13 mL of Buffer AW2 concentrate, as described on the bottle.
  b. Tick the check box on the label to indicate that ethanol has been added.
  c. Store reconstituted Buffer AW2 at room temperature (15°C–25°C).

***Note:*** Reconstituted Buffer AW2 is stable for up to 1 year or until the kit expiration date when stored at room temperature.

***Note:*** Always mix reconstituted Buffer AW2 by shaking before starting the procedure.

11. Viral DNA extraction
  Before starting, let the samples reach room temperature. All centrifugation steps were carried out at room temperature (15°C–25°C).
  a. Pipet 25 μL QIAGEN Protease into a 1.5 mL microcentrifuge tube
  b. Add the 200 μL of the VLPs sample into the microcentrifuge tube.
    ***Note:*** If the sample volume is less than 200 μL, add the appropriate volume of SM Buffer solution to bring the volume of the protease and the sample to a total of 225 μL.
  c. Add 200 μL Buffer AL. Close the cap and mix by pulse-vortexing for 15 s.
    ***Note:*** To ensure efficient lysis is essential that the sample and Buffer AL are mixed thoroughly to yield a homogeneous solution.
    **CRITICAL:** Do not add QIAGEN Protease directly to Buffer AL.
    **CRITICAL:** Buffer AL contains guanidine hydrochloride, forming highly reactive compounds when combined with bleach. Do not add bleach or acidic solutions directly to waste containing Buffer AL.
  d. Incubate at 56°C for 15 min in a heating block.
  e. Briefly centrifuge the 1.5 mL tube to remove drops from the inside of the lid.

12. Viral DNA purification
  Binding conditions are adjusted by adding ethanol to allow optimal binding of the viral DNA to the membrane. Lysates are then transferred onto a QIAamp MinElute column and viral nucleic acids are adsorbed onto the silica-gel membrane as the lysate is drawn through by centrifugation.
  a. Add 250 μL of ethanol (96–100%) to the sample, close the cap and mix thoroughly by pulse-vortexing for 15 s.
  b. Incubate the lysate with the ethanol for 5 min at room temperature (15°C–25°C).
    ***Note:*** If the ambient temperature exceeds 25°C, ethanol should be cooled on ice before adding to the lysate.
  c. Briefly centrifuge the 1.5 mL tube to remove drops from the inside of the lid.
  d. Carefully apply all lysates onto the QIAamp MinElute column without wetting the rim. Close the cap and centrifuge at 6000×*g* (8000 rpm) for 1 min.
  e. Place the QIAamp MinElute column in a clean 2 mL collection tube, and discard the collection tube containing the filtrate.
    ***Note:*** If the lysate has not entirely passed through the column after centrifugation, centrifuge again at a higher speed until the QIAamp MinElute column is empty.
  f. Carefully open the QIAamp MinElute column, and add 500 μL of Buffer AW1 without wetting the rim.
  g. Close the cap and centrifuge at 6000×*g* (8000 rpm) for 1 min.
  h. Place the QIAamp MinElute column in a clean 2 mL collection tube, and discard the collection tube containing the filtrate.
  i. Carefully open the QIAamp MinElute column, and add 500 μL of Buffer AW2 without wetting the rim.
  j. Close the cap and centrifuge at 6000×*g* (8000 rpm) for 1 min.
  k. Place the QIAamp MinElute column in a clean 2 mL collection tube, and discard the collection tube containing the filtrate.
  l. Carefully open the QIAamp MinElute column and add 500 μL of ethanol (96–100%) without wetting the rim.
  m. Close the cap and centrifuge at 6000×*g* (8000 rpm) for 1 min.
  n. Discard the collection tube containing the filtrate.
  o. Place the QIAamp MinElute column in a clean 2 mL collection tube. Centrifuge at full speed (20,000×*g*; 14,000 rpm) for 3 min to dry the membrane completely.
    ***Note:*** To evaporate any remaining liquid, it is recommended to place the QIAamp MinElute column into a new 2 mL collection tube (not provided), open the lid, and incubate the assembly at 56°C for 3 min to dry the membrane completely.
  p. Place the QIAamp MinElute column in a clean 1.5 mL microcentrifuge tube, and discard the collection tube with the filtrate.
  q. Carefully open the lid of the QIAamp MinElute column, and apply 30 μL of Buffer AVE or RNase-free water to the center of the membrane.
  r. Close the lid and incubate at room temperature for 1 min.
    ***Note:*** Incubating the QIAamp MinElute column loaded with Buffer AVE or water for 5 min at room temperature before centrifugation generally increases DNA yield.
  s. Centrifuge at full speed (20,000×*g*; 14,000 rpm) for 1 min.
  t. Eluted DNA can be collected in standard 1.5 mL microcentrifuge tubes.
    ***Note:*** Ensure that the elution buffer is at room temperature. If elution is done in small volumes (<50 μL), the elution buffer must be dispensed onto the center of the membrane for complete elution of bound DNA.
    ***Note:*** Elution volume is flexible and can be adapted according to the requirements of the downstream application. However, the recovered elution volume will be approximately 5 μL less than the volume of elution buffer applied onto the column.
    ***Note:*** If the purified viral DNA is used within 24 h, storage at 2°C–8°C. For periods longer than 24 h, storage at –20°C.
    ***Note:*** With the application of this protocol, we obtained an average of 160.7 ± 105.0 ng of total DNA from 200 μL of VLPs extracted from 250 mg of feces (Bikel et al., 2021).

### DNA library preparation and purification

**Timing: 1.5–2 hper sample**

In this step, the sequencing libraries of the extracted DNA from the VLPs are prepared using the Nextera XT DNA Library Preparation Guide (https://support.illumina.com/content/dam/illumina-support/documents/documentation/chemistry_documentation/samplepreps_nextera/nextera-xt/nextera-xt-library-prep-reference-guide-15031942-05.pdf). Prepare the sequencing libraries in a PCR laminar airflow cabinet to avoid contamination.

13. Assessing DNA Quality.
  a. Use an electrophoresis gel and a UV absorbance method to assess the quality of the DNA sample. Absorbance ratio values of 1.8–2.0 are considered adequate for this protocol.
14. Quantification of input DNA.
  a. To accurately quantify the input DNA and library concentration, use a fluorometric-based method such as Qubit dsDNA Assay.
  b. Dilute the sample DNA to a final concentration of 0.3 ng/μL in nuclease-free water.

**CRITICAL:** Avoid using DNA quantification methods that measure total nucleic acid content, such as Nanodrop or other UV absorbance methods. Contaminants such as ssDNA, RNA, and oligonucleotides are not substrates for the Nextera XT assay.

**CRITICAL:** A DNA concentration of 0.3 ng/μL is essential because the Nextera XT Protocol is optimized for ∼1 ng of input DNA contained in 5 μL. If the total concentration of DNA extracted from the VLP samples results low, an additional DNA concentration step might be needed.

***Note:*** Maintain the DNA sample on the ice during the process or store it at −20°C until use.

15. DNA Tagmentation.
  The Nextera XT transposase simultaneously fragments the input DNA and adds adapter sequences during this step. The reactions can be assembled in sterile PCR tubes.
  a. Thaw the diluted DNA sample and the following Kit reagents on ice.
    i. Amplicon Tagment Mix (ATM) and Tagment DNA Buffer (TD).
    ii. Maintain the Neutralize Tagment Buffer (NT) at room temperature.
  b. Vortex and visually inspect all reagents to make sure there is no precipitated. Suppose there is, vortex gently until the precipitate is resuspended.
    ***Note:*** Assemble the reaction in the order described for optimal kit performance. The reaction does not need to be assembled on ice.
  c. Add 10 μL TD buffer to a new PCR tube.
  d. Add 5 μL input DNA 0.3 ng/μL (1.5 ng total) to the tube with the TD buffer and gently pipette up and down five times to mix.
    **CRITICAL:** Use the same amount of input DNA for all libraries.
    ***Note:*** The user can assemble more than one library simultaneously. However, we recommend only preparing together libraries that belong to the same experimental group to avoid cross-contamination among tested groups.
  e. Add 5 μL ATM buffer to the reaction tube containing the TD buffer and the DNA. Gently pipette up and down five times to mix.
  f. Centrifuge the reaction tube at 280×*g* at room temperature for 1 min.
  g. Incubate the reaction tube in a thermal cycler at 55° for 5 min and then hold at 10°C. Maintain the thermal cycler lid heated during the incubation.
  h. When the incubation reaches 10°C, immediately add 5 μL of the NT buffer to the reaction tube to neutralize the reaction. Pipette up and down five times to mix.
    ***Note:*** To immediately neutralize the tagmentation reaction, adding the NT buffer while the reaction tube is still in the thermal cycler is more convenient.
  i. Centrifuge the reaction tube at 280×*g* at room temperature for 1 min.
  j. Maintain the samples at room temperature for 5 min.

16. PCR Amplification.
  During this step, the tagmented DNA is amplified for a limited number of PCR cycles to add the index i7 and i5.
  a. Select a pair of one i7 plus one i5 index primer for each library.
    **CRITICAL:** Two libraries cannot have the same pair of index primers, as they would be interpreted and sequenced as the same sample.
  b. Thaw the selected primers and the Nextera PCR Master Mix (NPM) on ice.
    i. After all reagents are thawed mix each tube by inverting 3–5 times and centrifuge at 280×*g* for 1 min.
  c. Add 15 μL of NPM to the tagmented DNA sample.
  d. Add 5 μL of index i7 and 5 μL of index i5 to each sample. Gently pipette up and down five times to mix.
    **CRITICAL:** Change tips between index primers to avoid cross-contamination.
  e. Centrifuge at 280×*g* at room temperature for 1 min.
  f. Perform PCR using the following program and maintain the lid heated during the process:

**Table:** 

PCR cycling conditions			
Steps	Temperature	Time	Cycles
Enzyme activation	72°C	3 min	1
Initial Denaturation	95°C	30 s	1
Denaturation	95°C	10 s	12 cycles
Annealing	55°C	30 s	
Extension	72°C	30 s	
Final extension	72°C	5 min	1
Hold	10°C	Forever

    **CRITICAL:** Use the same number of PCR cycles for all libraries to avoid over-estimation in a particular library. Adding extra cycles of PCR could produce low-quality sequence results.
    **Pause Point:** The user can safely stop before proceeding to PCR clean-up. The user can either keep the samples at 10°C overnight (12–14 h) inside the thermal cycler or store at 2°C–8°C for up to 2 days.

17. PCR Clean-Up.
  This step uses AMPure XP beads to purify the DNA library and removes short library fragments.
  ***Note:*** Before start, bring AMPure XP beads to room temperature.
  a. Vortex the AMPure XP beads to evenly disperse them.
  b. Add 30 μL of AMPure XP beads to each amplified library. Gently pipette up and down ten times to mix.
    ***Note:*** The volume of 30 μL of AMPure beads selects inserts of >500 bp. Refer to the Nextera XT DNA Library Preparation Guide to select the desired insert sizes.
  c. Incubate at room temperature for 5 min.
  d. Place the tube on a magnetic stand until the supernatant has cleared.
  e. While the tube is on the magnetic stand, carefully remove and discard the supernatant.
    ***Note:*** If any beads are aspirated, dispense back to the tube and wait until the supernatant is clear before removing again.
  f. While the tube is on the magnetic stand, add 200 μL of 80% ethanol to wash the beads.
    **CRITICAL:** Prepare fresh 80% ethanol from absolute ethanol. Ethanol can absorb water from the air altering the concentration and impacting the results.
  g. Maintain the tube on the magnetic stand and incubate for 30 s.
  h. Remove and discard the supernatant carefully.
  i. Maintain the tube on the magnetic stand and allow the beads to air-dry for 15 min.
    **CRITICAL:** Do not over-dry the beads. This could reduce the amount of library recovered.
  j. Remove any excess ethanol with a clean tip.
  k. Remove the tube from the magnetic stand and add 50 μL of nuclease-free water. Gently pipette up and down ten times to mix.
  l. Incubate at room temperature for 2 min.
  m. Place the tube on the magnetic stand and wait until the supernatant has cleared.
  n. While the tube is on the magnetic stand, transfer the supernatant carefully to a clean tube.
  o. Quantify the final library with a fluorometric-based method such as Qubit dsDNA assay, and check the size distribution in a High Sensitivity DNA Bioanalyzer.
  p. All final libraries can be pooled together and sequenced in any Illumina platform.
    ***Note:*** To achieve an even sequencing depth among all samples is essential that all libraries are pooled in equimolar proportions. As a reference, we adjusted each library to a final concentration of 2nM (Bikel et al., 2021).
    ***Note:*** The Nextera libraries contain Illumina adapters; thus, they could be sequenced in any Illumina platform. As a reference, we used the NextSeq500 system in a 2 × 150 pair-end mode and obtained 74,859,356 reads (an average of 2,673,548 reads per sample) (Bikel et al., 2021).
    ***Note:*** According to Illumina's recommendations, the sequencing read length for De novo sequencing ranges from 2 × 150 to 2 × 300 bp. As a reference, when sequencing with 2 × 150 bp, we assembled 18,602 viral contigs with ≥500 nt of length (Bikel et al., 2021).

## Expected outcomes

**Figure 2:**
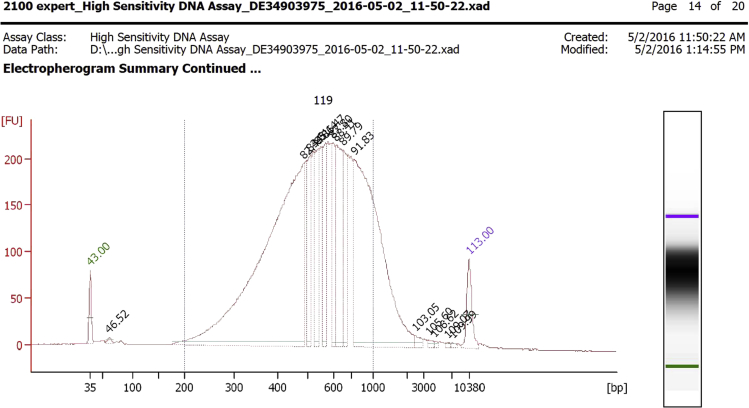
DNA bioanalyzer graph of a purified Nextera library built with 1.5 ng of viral DNA

The library preparation with 1.5 ng of input DNA yields ∼200 ng of library with a size distribution >500 bp (Figure 2).

## Quantification and statistical analysis

The data used for this protocol are in NCBI under BioProject accession number: PRJNA646512. In addition, all the scripts described below are in the Github repository https://github.com/lab8a/2021-iScience-Phageome.

### Cleaning and clustering of sequenced reads

1. First, perform the quality visualization with FastQC.

**CRITICAL:** Prior to the beginning and after the pretreatment, visualize the quality of the reads with FastQC. The most important parameters are: 'Per base sequence quality', 'Per sequence content', 'Per base GC content', 'Per base N content', 'Overrepresented sequences' and 'Adapter content'. All of these parameters must be checked as Passed after the pretreatment.

2. After that, remove the first 20 nucleotides (adapters) with Fastx Toolkit (adapter removal step).

>fastx_trimmer -v -f 20 -i Reads.fastq –o Reads_trimmed_First_20nt.fastq

3. Additionally, include the following steps: dereplication, remotion of adapters, and low-quality bases (PHRED Q30 score) using Trim_Galore.

> trim_galore -q 30 --paired --nextera R1-Reads_trimmed_First20nt.fastq R2- Reads_trimmed_First20nt.fastq # this line filter s the reads by quality using a quality threshold of ≥ 30 (Phred quality scores) and the Nextera sequencing adapters.

4. Then, remove potential human and bacterial contamination. To achieve this, use the BWA aligner against the Homo Sapiens GRCh38.p13 reference genome (GenBank: GCA_000001405.28) and the Kraken database against bacteria NR database using the default parameters.

>kraken --db krakenDB/bacteria_RefSeq --fastq-input --paired quality-reads-R1.fq quality-reads-R2.fq --threads 10 --unclassified-out Unclassified-Reads --classified-out Classified-Reads --output out.wdir

>bwa mem Hs_Genome-v38.fasta -t 7 -p interleaved-Unclassified-Reads.fq > Hs_aln_unclass.sam

5. Finally, cluster the quality-filtered reads at 95% identity using CD-HIT to generate a unique and non-redundant dataset. Then, remove those reads mapped against human and bacterial genomes in the previous steps, the remaining set is named quality-filtered reads.

***Note:*** The complete script for these steps is in the *CommandLines_GLL-iScience.md* file.

>samtools view -b -S -f 4 Hs_aln_unclass.sam > ViralReads.bam # extract unmapped reads

>bamToFastq -i ViralReads.bam -fq ViralReads.bam.fq # convert the the information in the .bam to a .fastq

>samtools view -b -S -f 4 Hs_aln_unclass.sam > ViralReads.bam # extract unmapped reads

>bamToFastq -i ViralReads.bam -fq ViralReads.bam.fq # convert the the information in the .bam to a .fastq

>grep -c "@N" ViralReads.fq > Ids_ViralReads_firts.txt # get all the unmapped ids-reads

>sort -u Ids_ViralReads_redundant.txt > Ids_ViralReads_final.txt

>seqtk subseq Unclassified-Reads.fq Ids_ViralReads_final.txt > Final-ViralReads.fq

>grep -c "@N" ViralReads.fq > Ids_ViralReads_firts.txt

>sort -u Ids_ViralReads_redundant.txt > Ids_ViralReads_final.txt

>seqtk subseq Unclassified-Reads.fq Ids_ViralReads_final.txt > Final-ViralReads.fq #using the unmapped ids-reads we collected the reads in pair mode

### Analysis of viral read richness

6. Determine viral richness between groups by collecting 1,000 random subsamples of 149,000 single-end quality-filtered reads using seqtk subseq (the number of reads is determined by the sample with the minor sequencing depth). Perform this exercise to simulate a rarefaction analysis without including a taxonomic bias on the data.

> for s in {1..1000}; do seqtk sample -s$s Final-All_ViralReads_H-10_R1.fq 149000 > sub_$s∖_H-10_149k_R1.fq; done # This is an example with one sample

7. Then, cluster each sub-sample at 95% identity with CD-HIT to identify the non-redundant and unique group of reads, and finally, count the reads per file.

>for s in sub_∗fq ; do cd-hit -i $s -o $s.cd-hit_95.id.fq -c 0.95 -M 0 -T 64 > $s.out; done # This is an example with one sample

>for s in ∗H-10∗cd-hit_95.id.fq ; do grep -c 'ˆ@NS' $s ; done | awk '{sum =+$1}END{print sum/1000}' # This is an example with one sample

***Note:*** The complete script for this method can be found in the *subseq_cdhit.md* file.

### Functional profiles and pVOGs analysis

8. Map quality filtered reads against the viral NR RefSeq and pVOGs databases using BLASTX with a maximum of 50 reported target sequences and a maximum e-value cutoff of 0.001.

>makeblastdb -in pVOGs.faa -dbtype prot -out pVOGs -parse_seqids

>for s in Final-All_ViralReads_∗id.fa; do blastx -db pVOGs -query $s -out $s∖pvogs.blastx -num_threads 64 -evalue 1e-3 -max_target_seqs 50 -outfmt 7; done

9. Then, generate a relative abundance matrix using an in-house bash script. Annotate this matrix according to the KEGG classification of each predicted protein and the UniProtKB online database using an in-house bash script. For the Prokaryotic Virus Orthologous Groups (pVOGs), map the quality-filtered reads against this database with a maximum e-value cutoff of 0.001 and 50 reported target sequences. Finally, generate the matrix with the pVOGs classification per sample using an in-house bash script. All these in-house bash developed scripts are in the reads_blastx_pvogs.md file.

### Classification of viral reads

10. Annotate the taxonomical classification to the quality-filtered and non-redundant sequences (unique) according to the Internationally Committee on Taxonomy of Viruses (ICTV). To this end, use BLASTX with a maximum e-value cutoff of 0.001 against the NR RefSeq viral database and obtain the Last Common Ancestor (LCA) with the MetaGenomeAnalyzer (MEGAN6) algorithm considering the following parameters: Min Support: 1, Min Score: 40.0, Max Expected: 0.01, Top Percent: 10.0, Min-Complexity filter: 0.44.

>makeblastdb -in viral.all.protein.single-line.faa -dbtype prot -out viral_refseq -parse_seqids

>for s in Final-All_ViralReads_∗id.fa; do blastx -db viral_refseq -query $s -out $s.blastx -num_threads 64 -evalue 1e-3; done

11. Calculate the relative abundance per sample using the absolute read count for selected viral taxa and the total reads from each sample. This script is in the *reads_blastx_megan.md* file.

### *De novo* contig assembly

12. Construct the de novo assembly using all the quality-filtered reads from all samples with the IDBA-UD assembler. However, due to a sample mixed-assembly, the chimeric contigs are filtrated only to obtain the contigs covering ≥80% of their total size by the viral reads in at least one sample.

> cat Final-ViralReads_R1∗.fq > Final-ViralReads_R1_allsamples.fastq # Merge all the R1 files

> cat Final-ViralReads_R2∗.fq > Final-ViralReads_R2_allsamples.fastq # # Merge all the R2 files

> paste Final-ViralReads_R1_allsamples.fastq Final-ViralReads_R2_allsamples.fastq | paste - - - - | awk -v OFS="∖n" -v FS="∖t" '{print($1,$3,$5,$7,$2,$4,$6,$8)}' > Interleave_ViralReads.fastq # Merge the R1 and R2 files in a interleaved fastq

> cat Interleave_ViralReads.fastq | sed -n '1∼4s/ˆ@/>/p;2∼4p' > Interleave_ViralReads.fasta # Convert from fastq to fasta format

> idba_ud -r Interleave_ViralReads.fasta --num_threads 8 --mink 20 --maxk 125 --pre_correction -o out.dir # De novo assembly with IDBA-UD assebler

13. Then, map each sample reads individually with Bowtie2 against the de novo viral assembly using the end-to-end mode with default parameters.

***Note:*** Select only the viral scaffolds covered >80% in length by the reads of at least one sample to discard chimeras. Remove the scaffolds with less than 4 kB to have a higher chance of only keeping nearly complete viral genomes. Lastly, use CD-HIT with a 95% clustering identity to eliminate the redundant scaffolds. These scripts are in the CommandLines_GLL-iScience.md file.

### Taxonomic classification of *de novo* assembly

14. Obtain the taxonomic classification of each scaffold using DC_MEGABLAST against the Nt NCBI viral genomes database with a maximum of 50 reported target sequences and a maximum e-value cutoff of 0.001.

>makeblastdb -in viral_refseq_nucl.fasta -dbtype db_nucl_refseq.faa -out db_nucl_refseq -parse_seqids

>blastn -task dc-megablast -db db_nucl_refseq -query FinalViralScaffolds_larger4Kb.fasta -out FinalViralScaffolds_larger4Kb.megablast.blastn -template_type coding_and_optimal -template_length 16 -evalue 1e-3 -num_threads 64

15. Assign the final taxonomy using the Last Common Ancestor (LCA) with the MetaGenomeAnalyzer (MEGAN6) algorithm considering the following parameters: Min Support: 1, Min Score: 40.0, Max Expected: 0.01, Top Percent: 10.0, Min-Complexity filter: 0.44. Map all the scaffolds without a taxonomical classification with DC_MEGABLAST using BLASTX against the NR NCBI viral protein database with the same above described parameters for DC_MEGABLAST.

>makeblastdb -in viral.all.protein.single-line.faa -dbtype prot -out viral_refseq -parse_seqids

>blastx -db viral_refseq -query FinalViralScaffolds_larger4Kb.fasta -out FinalViralScaffolds_larger4Kb.fasta.blastx -evalue 1e-3 -num_threads 64

16. Assign the final taxonomy of the BLASTX scaffolds with the LCA from MEGAN6, using the same parameters as described above for DC_MEGABLAST. These scripts are in the contigs_blastx_megan.md file. Finally, perform the VirSorter2 classification of each scaffold with the default parameters.

>virsorter run -w virsorter_FinalViralScaffolds_larger4Kb -i FinalViralScaffolds_larger4Kb.fasta -j 64

***Note:*** The virsorter.md contains the steps of this analysis.

17. Perform the last classification using BLASTX against the Prokaryotic Virus Orthologous Groups (pVOGs) database with a maximum e-value cut-off of 0.001 and maximum target sequences to report set to 50.

>for s in Final-All_ViralReads_∗id.fa; do blastx -db pVOGs -query $s -out $s∖pvogs.blastx -num_threads 64 -evalue 1e-3 -max_target_seqs 50 -outfmt 7; done

***Note:*** The script of this method can be consulted in the file *reads_blastx_pvogs.md*.

### Differential abundance of phage contigs

18. Use the recruitment of reads to the contigs assembly to construct an abundance matrix. Define the coverage from reads mapping (Bowtie2) at R90% identity and R80% length. Convert the mapping outputs into a normalized abundance matrix using an in-house R script using the Reads Per Kilobase per Million sequenced reads per sample (RPKM). The name of this script is multi_contingencty_table_transformations_taxa.R. The next line is an example of using this script.

>cat input_table.txt | Rscript multi_contingencty_table_transformations.R 10 0.5 test 20000

### Richness and diversity of phage contigs

19. Evaluate the contig assembly's richness and diversity based on the median of 10,000 rarefactions with a sequencing depth equal to the smallest sample based on the RPKM matrix. Conduct this process in QIIME 1.9. Assign the presence of phage contigs in the samples as either core phages: detected in >80% of the samples; common phages: in >50% and <80%; and individual phages: appearing in <50% of the population. These scripts are in the alpha_div_rarefaction.R and alpha_div_rar_compare.R files.

>cat input_table.txt | Rscript alpha_div_rar_compare.R 10000 output_prefix 149000 # To generate the rarefactions

>cat input_table.txt | Rscript alpha_div_rarefaction.R 50 output_prefix chao1 # To calculate the alpha diversity

### Bacteria and biochemical parameters correlations

20. Calculate the correlations using the Spearman coefficient with rcorr function in R. Select the RPKM matrix for the contigs phage abundance and the relative frequency of the significant over-abundant taxa for O and OMS for the microbiota abundance. The script used for this step is in the spearman_corr.md file. The following is an example of use.

>Rscript spearman_correlations.R input_table.txt spearman_rho.txt spearman_p-value.txt

## Limitations

A significant limitation for better understanding the role of the human gut virome in health and disease is the lack of standardized methods that allow high throughput virome analysis, coupled with the lack of a universal viral marker, unlike the 16S gene in the bacteriome. Therefore, studying the virome requires large-scale metagenomic sequencing approaches.

The first challenge in this study is the number of samples, which probably accounted for the lack of statistical significance obtained in some of the analyses.

We used the TAG method that strongly selects against ssDNA templates for library preparation, obtaining ssDNA viruses near or below detection limits. However, to eliminate the bias due to the TAG method, we decided to eliminate from the analysis all ssDNA viruses. Further improvements in sequencing library preparation techniques are essential to overcome this bias and bring metagenomic research of human gut phageome to a fully quantitative level.

The vast majority of contigs assembled from the data generated in this study could not be aligned to any known viral genomes in the NCBI RefSeq database, suggesting that many of the sequences are of unknown viral origin. In this regard, more virome studies are needed to gain a broader understanding of the composition of the human gut virome.

We performed an accurate estimation of viral community composition and diversity. However, we should note that our assemblies may represent fragments of the same phage genome that could affect accurate estimates about our phageome and its prevalence in the human population.

## Troubleshooting

### Problem 1

The filters clog very quickly with bacterial debris (step 2).

### Potential solution

Increase the time used for centrifugation.

### Problem 2

The filter was broken (step 2).

### Potential solution

Change the filter for a new one and discard the old one.

### Problem 3

Fluorescence is not observed in the microscope (step 6).

### Potential solution

Prepare a new staining reaction and immediately observe after slide preparation.

### Problem 4

In a few instances, view fields in TEM were obstructed by extensive, amorphous, dark-staining material (step 7).

### Potential solution

One of the solutions for this problem is to increase the number of washes with the Amicon Ultra-15 Centrifugal Filter Units 100 KDa after all the filtrate is processed. To this end, add SM Buffer to the top chamber of the Amicon and spin at 5000 g for 2–5 min to wash the filtrate. Eight washes of the Amicon allow us a more precise visualization of viral particles by electron microscopy (TEM).

## Resource availability

### Lead contact

Further information and requests for resources and reagents should be directed to and fulfilled by the lead contact, Adrian Ochoa-Leyva (adrian.ochoa@ibt.unam.mx).

### Materials availability

This study did not generate new unique reagents.

## Data Availability

All original code have been deposited to GitHub: https://github.com/lab8a/2021-iScience-Phageome and in Zenodo: https://doi.org/10.5281/zenodo.5846703. The accession number for the sequenced data reported in this paper is NCBI BioProject: PRJNA646512. Accession numbers are also listed in the key resources table. Any additional information required to reanalyze the data reported in this paper is available from the lead contact upon request.

## References

[bib1] Altschul S.F., Gish W., Miller W., Myers E.W., Lipman D.J. (1990). Basic local alignment search tool. J. Mol. Biol..

[bib2] Bikel S., López-Leal G., Cornejo-Granados F., Gallardo-Becerra L., García-López R., Sánchez F., Equihua-Medina E., Ochoa-Romo J.P., López-Contreras B.E., Canizales-Quinteros S. (2021). Gut dsDNA virome shows diversity and richness alterations associated with childhood obesity and metabolic syndrome. iScience.

[bib3] Caporaso J.G., Kuczynski J., Stombaugh J., Bittinger K., Bushman F.D., Costello E.K., Fierer N., Pẽa A.G., Goodrich J.K., Gordon J.I. (2010). QIIME allows analysis of high-throughput community sequencing data. Nat. Methods.

[bib4] Fu L., Niu B., Zhu Z., Wu S., Li W. (2012). CD-HIT: accelerated for clustering the next-generation sequencing data. Bioinformatics.

[bib5] Gallardo-Becerra L., Cornejo-Granados F., García-López R., Valdez-Lara A., Bikel S., Canizales-Quinteros S., López-Contreras B.E., Mendoza-Vargas A., Nielsen H., Ochoa-Leyva A. (2020). Metatranscriptomic analysis to define the Secrebiome, and 16S rRNA profiling of the gut microbiome in obesity and metabolic syndrome of Mexican children. Microb. Cell Factories.

[bib6] Guo J., Bolduc B., Zayed A.A., Varsani A., Dominguez-Huerta G., Delmont T.O., Pratama A.A., Gazitúa M.C., Vik D., Sullivan M.B. (2021). VirSorter2: a multi-classifier, expert-guided approach to detect diverse DNA and RNA viruses. Microbiome.

[bib7] Hoyles L., McCartney A.L., Neve H., Gibson G.R., Sanderson J.D., Heller K.J., van Sinderen D. (2014). Characterization of virus-like particles associated with the human faecal and caecal microbiota. Res. Microbiol..

[bib8] Huson D.H., Auch A.F., Qi J., Schuster S.C. (2007). MEGAN analysis of metagenomic data. Genome Res..

[bib9] Huson D.H., Albrecht B., Bağci C., Bessarab I., Górska A., Jolic D., Williams R.B.H. (2018). MEGAN-LR: new algorithms allow accurate binning and easy interactive exploration of metagenomic long reads and contigs. Biol. Direct.

[bib10] Jepson C.D., March J.B. (2004). Bacteriophage lambda is highly stable DNA vaccine delivery vehicle. Vaccine.

[bib11] Jończyk E., Kłak M., Międzybrodzki R., Górski A. (2011). The influence of external factors on bacteriophages—review. Folia Microbiologica.

[bib12] Kim M.S., Park E.J., Roh S.W., Bae J.W. (2011). Diversity and abundance of single-stranded DNA viruses in human feces. Appl. Environ. Microbiol..

[bib21] Kristensen D.M., Waller A.S., Yamada T., Bork P., Mushegian A.R., Koonin E.V. (2013). Orthologous gene clusters and taxon signature genes for viruses of prokaryotes. J. Bacteriol..

[bib13] Langmead B., Salzberg S.L. (2012). Fast gapped-read alignment with Bowtie 2. Nat. Methods.

[bib14] Li H., Durbin R. (2010). Fast and accurate long-read alignment with Burrows-Wheeler transform. Bioinformatics.

[bib15] Love M.I., Huber W., Anders S. (2014). Moderated estimation of fold change and dispersion for RNA-seq data with DESeq2. Genome Biol..

[bib16] Mullan W.M.A. (2001). Isolation and Purification of Bacteriophages. https://www.dairyscience.info/index.php/isolation-and-purification-of-bacteriophages.html.

[bib17] Peng Y., Leung H.C.M., Yiu S.M., Chin F.Y.L. (2012). IDBA-UD: a de novo assembler for single-cell and metagenomic sequencing data with highly uneven depth. Bioinformatics.

[bib18] Rho M., Tang H., Ye Y. (2010). FragGeneScan: predicting genes in short and error-prone reads. Nucleic Acids Res..

[bib19] Shkoporov A.N., Clooney A.G., Sutton T.D.S., Ryan F.J., Daly K.M., Nolan J.A., McDonnell S.A., Khokhlova E.V., Draper L.A., Forde A. (2019). The human gut virome is highly diverse, stable, and individual specific. Cell Host and Microbe.

[bib20] Schindelin, J., Arganda-Carreras, I., Frise, E., Kaynig, V., Longair, M., Pietzsch, T., Preibisch, S., Rueden, C., Saalfeld, S., Schmid, B., el at. 2012. Fiji: an open-source platform for biological-image analysis. *Nat. Methods*, *9*, 676–682. 10.1038/nmeth.2019. PMC3855844 22743772

